# Multiple-Time-Scale Analysis of Attention as Revealed by EEG, NIRS, and Pupil Diameter Signals During a Free Recall Task: A Multimodal Measurement Approach

**DOI:** 10.3389/fnins.2019.01307

**Published:** 2019-12-06

**Authors:** Takashi Numata, Masashi Kiguchi, Hiroki Sato

**Affiliations:** ^1^Center for Exploratory Research, Research & Development Group, Hitachi, Ltd., Saitama, Japan; ^2^Department of Bioscience and Engineering, Shibaura Institute of Technology, Saitama, Japan

**Keywords:** multimodal measurement, electroencephalogram, near-infrared spectroscopy, pupil diameter, multiple-time-scale analysis, free recall task, serial position effect

## Abstract

Attention plays a fundamental role in acquiring and understanding information. Therefore, it is useful to evaluate attention objectively in such fields as education and mental health. Aimed at extracting objective indicators of attention from physiological signals, this study examined the characteristics of electroencephalography (EEG), near-infrared spectroscopy (NIRS), and pupil diameter signals during a free recall task. The objective was to clarify the temporal characteristics of these signals in relation to attention. We used a free recall task as a cognitive task with an attentional load. The participants attempted to memorize and then recall 13 serially presented words. Our hypothesis was that the significant physiological responses should differ depending on the time scale of the attention evaluation. The physiological responses were compared on the basis of differences between success and failure to recall a word on a short time scale, in terms of the attentional state among five serial position groups on a middle time scale, and on the basis of differences between trials with many and few words recalled on a long time scale. We found that the response of each physiological signal depended on the attention in the different time-scale comparisons. (1) The P300 amplitudes of the EEG signals for the words that were recalled were significantly higher than those for the words that were not recalled. (2) Pupillary dilation differed significantly depending on the serial position group. (3) Functional connectivity in the right hemisphere revealed by NIRS was significantly stronger in trials with many words recalled than in those with few words recalled. Different temporal characteristics of physiological signals with respect to attention were suggested by multimodal measurement and multiple-time-scale analysis. Consideration of these characteristics should help in the development of applications requiring objective attention evaluation.

## Introduction

Attention is a cognitive process that involves acquiring and understanding information. The quantity and quality of information acquired and the degree of understanding depend on the strength of the attention. Since the strength depends on the person’s current state and the external environment, practical applications and techniques for sharpening attention have been developed in various fields, including education (Schmidt, 1995; Lin, 2011), advertising (Pechmann and Stewart, 1990), marketing (Pieters et al., 2002), and mental health (Schmidt et al., 2009; Tamm et al., 2010). For example, learning with animated instructions, questions, and feedback can enhance a learner’s attention and improve understanding of the learned contents as revealed by test scores (Lin, 2011). In the mental health field, attention training has been used for treating mental disorders such as attention-deficit/hyperactivity disorder and social anxiety disorder (Schmidt et al., 2009; Tamm et al., 2010). To develop effective interventions and techniques for improving attention, it is important to evaluate attention objectively because doing so enables the effects of interventions and techniques to be quantified and attention to be sharpened appropriately on the basis of the person’s current attention.

Attention can be objectively evaluated by using physiological signals related to brain activity. Since attention is related to a variety of brain functions (attention function) (Posner and Rothbart, 2007), it is reflected in a variety of physiological signals such as brain electrical activity signals (electroencephalographic signals) measured by electroencephalography (EEG) (Fan et al., 2007; Neuhaus et al., 2010; Zhang et al., 2010) and cerebral blood volume signals measured by functional magnetic resonance imaging (fMRI) or near-infrared spectroscopy (NIRS) (Fan et al., 2005; Hagen et al., 2014). These physiological signals have been used to evaluate attention in previous studies (Fan et al., 2005, 2007; Azizian and Polich, 2007; Neuhaus et al., 2010; Zhang et al., 2010; Hagen et al., 2014; Nogueira et al., 2015). In addition, Pupil diameter is an indicator of the activity in the locus coeruleus (Sterpenich et al., 2006; Gilzenrat et al., 2010), and the locus coeruleus is the main hub of the brain’s noradrenergic system and it is thought to modulate the operations of the attentional systems (Posner and Rothbart, 2007; Alnæs et al., 2014; Unsworth and Robison, 2017).

From a practical application perspective, EEG signals, NIRS signals, and pupil diameter are useful physiological signals. These signals are particularly advantageous because they enable attention to be evaluated objectively on the basis of brain activity using non-invasive and low-restriction measurement.

Although attention has been evaluated by using physiological signals in previous studies (Fan et al., 2005, 2007; Azizian and Polich, 2007; Neuhaus et al., 2010; Zhang et al., 2010; Hagen et al., 2014; Nogueira et al., 2015), the characteristics of EEG, NIRS, and pupil diameter signals in relation to attention have not yet been fully clarified. In particular, the temporal characteristics of the responses of these physiological signals (physiological responses) in relation to attention are not well understood. In other words, characteristics of relationship between the physiological responses and attention in various time scale have not yet understood. Since physiological responses are obtained from different physiological phenomena and are induced by different physiological mechanisms, their temporal characteristics should differ, so the appropriate physiological responses should also differ depending on the time scale of the attention evaluation. In addition, these responses were induced by various physical and mental factors, and they reflect various cognitive processes. Therefore, it is not clear which response dominantly reflect the strength of attention in each time scale. For example, when information is serially presented for a non-negligible time, it is not clear whether it is better to evaluate the average strength of attention during the presentation by using averaged instantaneous physiological responses or slower physiological responses. In other words, it is not clear whether it is better to evaluate the averaged event-related potentials (ERPs) in EEG signals (Azizian and Polich, 2007; Neuhaus et al., 2010; Nogueira et al., 2015), pupillary responses in pupil diameter signals, or cerebral blood volume measured by NIRS. Thus, it is important to understand the temporal characteristics of the physiological responses so that an appropriate physiological signal can be selected for use in applications and techniques requiring attention evaluation. Multimodal measurement and multiple-time-scale analysis of physiological responses are effective for clarifying the temporal characteristics.

A free recall task is useful as an attention task for evaluating attention on multiple time scales. The free recall task is typically a word memory task in which the participant attempts to memorize serially presented words and then recall them in any order. The recall success rate for a word is affected by the word’s position in the series: the rates for early and late position words tend to be higher than average while those for mid-series words tend to be lower (Sederberg et al., 2006; Azizian and Polich, 2007; Serruya et al., 2014). The results of previous studies indicate that this serial position effect reflects the operation of a single system that is modulated by allocation of attention and is less vulnerable to interference than two distinct memory systems (Azizian and Polich, 2007). From findings of previous studies, a resource of domain-general central attention is limited, and attentional load and working memory recall has a linear relationship in the task (Camos et al., 2018; Loaiza and Halse, 2019). In addition, it is also suggested that processing and storage of memory rely on a shared pool of attentional resources (Jones, 1976; Camos et al., 2018; Loaiza and Halse, 2019). Therefore, attention can be evaluated by using memory retrieval performance in the task. In other words, attention has a mediating effect on enhanced encoding, and attention linked to encoding produces better recall performance for words presented in the initial position than for those presented in subsequent positions. Accordingly, EEG indices have been compared on the basis of task score (recalled performance in a free recall task) and serial position (Sederberg et al., 2006; Azizian and Polich, 2007; Serruya et al., 2014). For example, Azizian et al. used ERPs to demonstrate an attentional gradient in a serial position memory curve (Azizian and Polich, 2007). However, these studies used only EEG measurement and single-time-scale analysis (Sederberg et al., 2006; Azizian and Polich, 2007; Serruya et al., 2014).

In this study, we hypothesized that the significant physiological responses should differ depending on the time scale of the attention evaluation. To test this hypothesis, we performed multimodal measurement of EEG, NIRS, and pupil diameter signals during a free recall task and used multiple-time-scale analysis to clarify the temporal characteristics of the physiological responses related to attention. The physiological responses were evaluated on the basis of the differences between success and failure to recall a word on a short time scale, in terms of attention among five serial position groups comprised of various numbers of 13 words on a middle time scale, and on the basis of differences between trials with many and few words recalled on a long time scale. Clarifying these characteristics will contribute to the development of applications requiring objective attention evaluation.

## Materials and Methods

### Experimental Procedures

Ten healthy Japanese volunteers (nine male and one female) with a mean age of 40.7 ± 10.4 years participated in our experiment. Data from the participants were obtained in accordance with the standards of the internal review board of the Research & Development Group, Hitachi, Ltd., following receipt of written informed consent.

**FIGURE 1:**
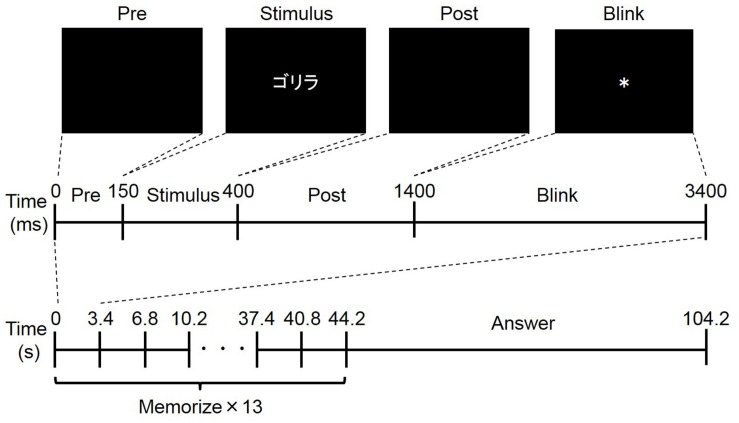
Overview of free recall task. The upper four figures show an example of word presentation. Word was presented in Japanese (“
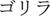
” means “gorilla,”). The middle figure shows the timeline of word presentation: pre-phase, stimulus phase, post-phase, and blink phase. The bottom figure shows the timeline of one trial composed of memorize and answer periods.

In the experiment, a free recall task was performed (Figure 1). Each participant performed ten trials, and each trial consisted of a memorization period and an answer period. In the memorization period, the participant was told to memorize as many words as possible, and 13 words were presented serially. The presentation of each word consisted of four phases: pre, stimulus, post, and blink. In the pre- and post-phases, a black background was presented for 150 ms and 1000 ms, respectively. In the stimulus phase, a word was presented for 250 ms. In the blink phase, an asterisk (^∗^) was presented for 2000 ms. Because blinking can induce a large noise in the EEG and pupil diameter signals (Nogueira et al., 2015), the participants were told not to blink except during the blink phase. The total duration for word presentation was 3400 ms, so the duration of the memorization period was 44.2 s. In the subsequent answer period, the participants were told to recall as many words as possible and to write them on a piece of paper. The duration of the answer period was 60 s, so the duration of a trial was 104.2 s. The 130 words used in the ten trials for each participant were randomly selected from among 260 Japanese words in a katakana database (Amano and Kondo, 1999). Each of the 260 words satisfied three conditions: had three morae, had more than 6.0 familiarity in the database, and were not a word for food. Before the experiment, each participant performed at least three trials using a different set of words to become familiar with the experimental procedure, particularly the restriction on blinking.

**FIGURE 2:**
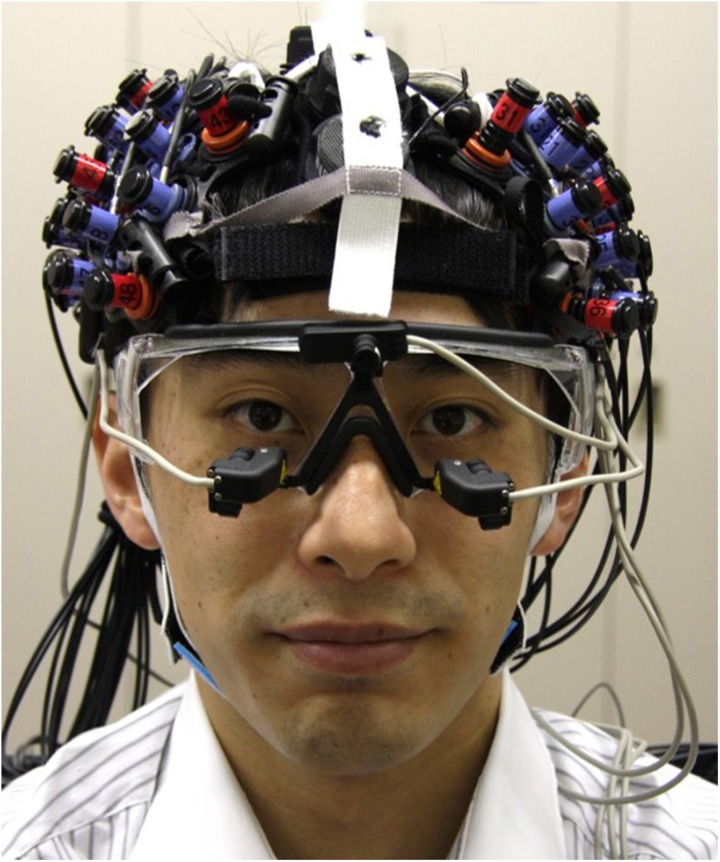
Multimodal measurement of EEG, NIRS, and pupil diameter signals. EEG electrodes were set on midline of head, and NIRS probes were set on both sides of head. Goggles with two cameras for measurement of pupil diameter were worn under electrodes and probes.

During task performance, the EEG signal was measured using a headset with EEG electrodes (DSI-7-Flex, Wearable Sensing); the change in the oxy-Hb concentration signal was measured using NIRS probes (ETG-7100, Hitachi Medical), and the pupil diameter signal was measured using goggles (EMR-9, NAC) fitted with two cameras (Figure 2). The sampling rates were 300, 10, and 60 Hz, respectively. These signals were synchronized with the task by sending a signal from the stimulus presentation computer at the beginning of each trial.

For the EEG measurement, three dry electrodes were set at the Fz, Cz, and Pz positions while reference electrodes were set at the A1 and A2 positions in accordance with the international 10–20 method of electrode placement (Sharbrough et al., 1991; Bell et al., 2008; Bin et al., 2009). For the NIRS measurement, 3 × 5 probes (22 channels) were set on the left and right frontal-parietal regions of the head. The probes were accurately set to match the F3 and F4 positions, which are near the front close to the midline, and so as not to interfere with the EEG electrodes and the goggles for pupil diameter measurement. For pupil diameter measurement, goggles with two cameras focused on the eyes were worn under the EEG electrodes and NIRS probes. The goggles had only partial lenses so as not to interfere with the wearing of eyeglasses. To minimize changes in the positions of the EEG electrodes and NIRS probes and noise induced by head motion, we fabricated headgear made of elastic bands that combined the electrodes and probes (Figure 2). Since the lengths of the bands could be adjusted, the headgear was easily adjusted to each participant’s head size and shape.

### Signal Processing

Three types of physiological responses were obtained from the multimodal measurement of physiological signals: P300 component from the EEG measurement, functional connectivity from the NIRS measurement, and pupil diameter change from the pupil diameter measurement. These physiological responses were extracted by using MATLAB R2017b (MathWorks, Inc.).

ERPs in EEG signals are one of the responses to tasks and can be observed during attention tasks including a free recall task (Azizian and Polich, 2007). Azizian et al. (Azizian and Polich, 2007) reported that a P300, which is a positive ERP, was observed in a free recall task after word presentation. In this study, peak-to-peak analysis was used to extract the amplitude of the P300 component (P300 amplitude) in the EEG signal in accordance with previous studies (Rosenfeld et al., 2004; Meijer et al., 2007). First, the EEG signals were filtered using a bandpass filter (0.003–50 Hz). Next, each signal from pre- to post-phase (1.4 s) was extracted as “word stimulus data.” To avoid the effect of noise, word stimulus data including blinking or body motion were excluded. Blinking was detected using the pupil diameter signals, and body motion was detected using the EEG signals. An instantaneous change of more than 30 μV was considered to be body motion. Baseline correction was applied to the data for each word stimulus by subtracting the average value of the 150-ms pre-phase before word onset. Finally, each P300 amplitude was extracted by subtracting the maximal value from the minimal value between 150 ms and 550 ms in the data for each word stimulus. The P300 amplitudes for the Fz, Cz, and Pz channels were extracted and compared on the basis of the differences in task score and serial position. In addition, spectral power bands in EEG signals can be also used to evaluate longer periods of attention by using the spectral analysis (Dockree et al., 2007; Romei et al., 2008). As the spectral analysis, alpha (8–14 Hz), beta (15–30 Hz), and gamma (30–50 Hz) frequency bands power and their ratios were calculated by using fast Fourier transform with hamming window, after trials with large artifacts were rejected.

There are strong correlations among NIRS signals in the low-frequency range (Sasai et al., 2012), reflecting the functional connectivity in brain networks in the same way as among blood oxygen-level dependent (BOLD) signals measured by fMRI (Sasai et al., 2012; Tong et al., 2012). Since attention function is related to the frontal and parietal brain regions of the brain, we calculated the functional connectivity corresponding to these regions to evaluate the differences among task scores for all words and trials and serial position groups. First, the NIRS signals recorded during the memorization periods were extracted as “trial data.” Next, the portions of the trial data corresponding to body motion (instantaneous change >0.4 mM mm) were excluded. Then, the remaining trial data were filtered with a bandpass filter (0.04–0.10 Hz). Finally, functional connectivity was extracted by calculating 44 × 44 Pearson cross-correlation coefficients among the NIRS signals in the trial data. We set three regions of interest as the physiological responses: the coefficients for the channels in the left hemisphere, those in the right hemisphere, and those between a channel in the left hemisphere and a channel in the right hemisphere (inter hemisphere). The NIRS signals were processed using the Platform for Optical Topography Analysis Tool (POTATo) (Sutoko et al., 2016).

The response of the left pupil diameter to each word stimulus was extracted. First, the word stimulus data were extracted in the same way as for the EEG signals. Data corresponding to blinking (instantaneous change >1.0 mm) were excluded. Then, the average pupil diameter during the pre-phase was derived as a baseline, and the data for each word stimulus was normalized using the baseline (Wierda et al., 2012):

$$P_{\text{norm}}=\frac{P_{\text{data}}-P_{\text{baseline}}}{P_{\text{baseline}}},$$

where *P*_*norm*_ is the normalized data, *P*_*data*_is the word stimulus data, and *P*_*baseline*_ is the baseline. In a previous study, subtractive baseline correction is recommended to relatively avoid the effect of small pupil size due to eye blinks and/or data loss (Mathôt et al., 2018). However, because the position of goggle was slightly adjusted not to interfere EEG electrodes and NIRS probes, baseline pupil diameter and amplitudes of pupillary changes were highly varied among participants. Therefore, we applied the combination of the subtractive baseline correction and divisive baseline correction, as well as the previous study (Wierda et al., 2012). The extracted pupil diameter changes were compared on the basis of the differences in task score and serial position.

### Multiple-Time-Scale Analysis

Using the physiological responses, we applied multiple-time-scale analysis of attention on the basis of the differences in task score and serial position. As mentioned, we hypothesized that the significant physiological responses should differ depending on the time scale of the attention evaluation. We thus evaluated the physiological responses for three time scales. More specifically, we evaluated physiological responses on the basis of differences between success and failure to recall a word (“recall word” and “fail word” on a short time scale), in terms of the attention among five serial position groups (on a middle time scale), and on the basis of differences between trials with many and few words recalled (“good recall trials” and “poor recall trials” on a long time scale). In other words, the task score was evaluated by success or failure to recall a word on the short time scale, and trials with many and few words recalled on the long time scale, and the serial position was used by five serial position groups on a middle time scale. Spectral power bands and their ratios were evaluated on the basis of only the differences between good and poor recall trials. Since it takes several seconds to observe the response of cerebral blood volume and the response is relatively broad (Aoki et al., 2011; Ogawa et al., 2014), the analysis duration of the NIRS signals included blink phase with a lag of one word presentation (3.4 s) and the last words in trials were excluded from evaluation on the short- and middle-time-scale analysis. These multiple-time-scale analysis and statistical analysis were also performed by using MATLAB R2017b (MathWorks, Inc.).

For the short-time-scale analysis, a “recall word” was a word the participant successfully recalled while a “fail word” was a word the participant failed to recall. Since the analysis duration of the EEG and pupil diameter signals was 1.4 s, comparison based on words reflects an attention span of about 1 s.

For the middle-time-scale analysis, the 13 words in the trial were divided into five serial position groups (1st–3rd words, 4th–6th words, 7th word, 8th–10th words, and 11th–13th words), and the average values of the physiological responses were compared among the groups. Comparison based on serial position group reflects an attention span of about 10 s. In addition, to confirm the previous findings on the effect of the serial position group on the task score, the success rates for the serial position groups were extracted. The success rate was the ensemble average across all participants.

For the long-time-scale analysis, a threshold was set for separating good recall trials from poor recall trials. The threshold was defined as the median number of recall words for the ten trials for each participant. The trials with a greater number of recall words than the threshold were regarded as good ones, and those with a smaller number were regarded as poor ones. The average values of the physiological responses between good and poor recall trials were then compared. This comparison reflects the attention span during the memorization period (44.2 s).

The success rates among serial position groups were compared using a one-way repeated-measures ANOVA (analysis of variance). The physiological responses on the short and long time scales were compared using a paired *t*-test for each channel or region, and those on the middle scale were compared using a one-way repeated-measures ANOVA. For the physiological responses reflected in the NIRS signals, the *z*-value of the correlation coefficient was compared as a measure of functional connectivity by using z transformation. Multiple corrections were performed using the false discovery rate. The statistical significance level was set at *p* < 0.05.

## Results

### Response to Serial Position Task

#### Success Rate

**FIGURE 3:**
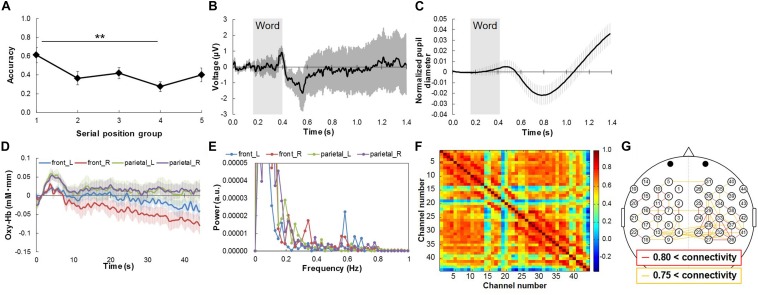
Word recall success rates and representative results of average physiological responses during free recall task. **(A)** Success rate for serial position groups. **(B)** Response waveforms of EEG for Cz channel, and **(C)** left pupil diameter change during task. Gray rectangles represent duration of word presentation. **(D)** Response waveforms of NIRS signals at front-left [front_L; channel no. 1 in **(F)**], front-right (front_R; no. 26), parietal-left (parietal_L; no. 9), and parietal-right channels (parietal_R; no. 27) during memorize phase. **(E)** Power spectral density of response waveforms of NIRS signals at front-left [front_L; channel no. 1 in **(G)**], front-right (front_R; no. 26), parietal-left (parietal_L; no. 9), and parietal-right channels (parietal_R; no. 27) during memorize phase. **(F)** Functional connectivity map during memorize phase. **(G)** High functional connectivity map with channel position of NIRS signals. Red and yellow lines represent channel pairs with correlation coefficient higher than 0.80 and between 0.75 and 0.80, respectively. Bars in **(A–D)** represent standard errors. ^∗∗^*p* < 0.01.

Figure 3 shows the word recall success rates and representative results for average physiological responses during the free recall task. The success rates were relatively high at first, then decreased (Figure 3A). One-way repeated ANOVA showed that the success rates differed significantly among serial position groups: *F*(2.512, 22.605) = 5.671, *p* < 0.05. *Post hoc* testing revealed that the success rate for the first serial position group was significantly better than that for the fourth one (*p* < 0.01). This means that the serial position group affects the task score.

#### Physiological Responses

Regarding the physiological responses, P300 was observed in the EEG signals for all channels after word presentation (Figure 3B). The pupil diameter decreased and then greatly increased after word presentation (Figure 3C) [Although it seems to show an increase first, the increase was not significant in comparison with the pupil diameter at the baseline (0s)]. We thus extracted two physiological responses from the pupil diameter signals by peak-to-peak analysis: the amplitude of the decrease (“pupillary contraction”) and that of the increase after pupillary contraction (“pupillary dilation”). The amount of pupillary contraction was defined as the difference between the minimal pupil size and the maximal pupil size after the beginning of word presentation (0.15 s in Figure 3C) and before the decrease in pupil diameter. Pupillary dilation was defined as the difference between the minimal pupil size and the maximal pupil size after the decrease in pupil diameter. The NIRS signals initially increased and then oscillated during the memorization phase (Figure 3D). Since a clear fluctuation which matched to the frequency of word presentation (0.29 Hz) was not found in the NIRS signals by a spectral analysis using fast Fourier transform with hamming window (Figure 3E), functional connectivity should be effective for multiple-time-scale analysis rather than peak analysis. Regarding functional connectivity in the NIRS signals, correlation coefficients of more than 0.8 (red lines in Figure 3G) were observed for the left and right hemispheres while ones of less than 0.8 (only orange lines in Figure 3G) were observed for the inter hemisphere (Figures 3F,G).

### Comparison of Physiological Responses by Multiple-Time-Scale Analysis

**FIGURE 4:**
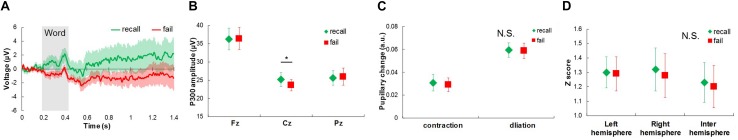
Physiological responses extracted from EEG and pupil diameter signals between recalled words and failed words in short time-scale comparison. **(A)** Average response waveforms of EEG signals for Cz channel for recall words (recall) and fail words (fail). Gray rectangle represents duration of word presentation. **(B)** Comparison of P300 amplitude between recall and fail words. **(C)** Comparison of pupillary contraction and dilation between recall and fail words. **(D)** Comparison of functional connectivity between recall and fail words. Bars represent standard errors. N.S., not significant. ^∗^*p* < 0.05.

**FIGURE 5:**
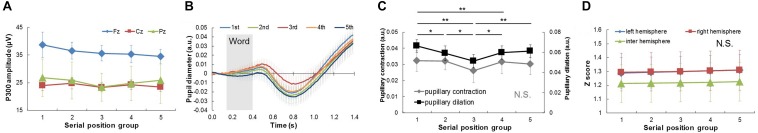
Physiological responses extracted from EEG and pupil diameter signals among five serial position groups in middle time-scale comparison. **(A)** Comparison of P300 amplitude among serial position groups. **(B)** Average response waveforms of pupil diameter signals for five serial position groups. Gray rectangle represents duration of word presentation. **(C)** Comparison of pupillary contraction and dilation among serial position groups. Bars represent standard errors. **(D)** Comparison of functional connectivity among serial position groups. N.S., not significant. ^∗^*p* < 0.05 and ^∗∗^*p* < 0.01.

**FIGURE 6:**
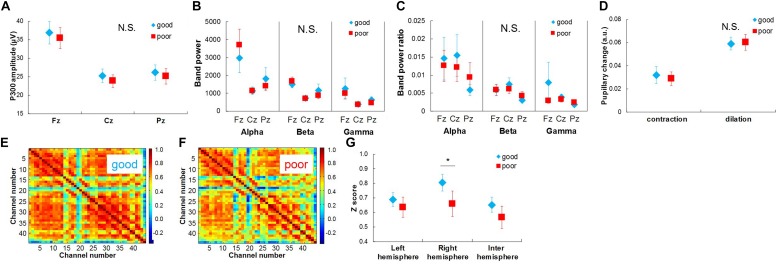
Physiological responses extracted from EEG, NIRS, and pupil diameter signals between well recalled trials and poorly recalled trials in long time-scale comparison. **(A)** Comparison of P300 amplitude between well recalled trials (high) and poorly recalled trials (low). **(D)** Comparison of pupillary contraction and dilation between well recalled trials (high) and poorly recalled trials (low). **(E)** Average functional connectivity map of NIRS signals in well recalled trials and **(F)** poorly recalled trials. **(G)** Comparison of z scores of functional connectivity between well and poorly recalled trials. Bars in **(A–D)** and **(G)** represent standard errors. N.S., not significant. ^∗^*p* < 0.05.

Figures 4–6 show the results of our multiple-time-scale comparison of the physiological responses based on task score and serial position. Significant differences in physiological responses were observed between different time scales depending on the modality.

In the short-time-scale comparison (Figure 4), the P300 amplitude for the Cz channel for the recall words was significantly higher than that for the fail words: *t*(9) = 3.269, *p* < 0.05 (Figures 4A,B). Pupillary changes and functional connectivity did not show any significant differences (Figures 4C,D).

In the middle-time-scale comparison (Figure 5), one-way repeated ANOVA of the pupillary change with the effect of pupillary dilation and serial position group revealed a significant interaction effect: *F*(3.217, 28.952) = 14.635, *p* < 0.001. *Post hoc* testing revealed that pupillary dilation for the first serial position group was significantly larger than those for the second, third, and fourth ones (third: *p* < 0.001; second and fourth: *p* < 0.05), and pupillary dilation for the third serial position group was significantly smaller than those for the other groups (first: *p* < 0.001; second, fourth, and last: *p* < 0.05) (Figures 5B,C). The P300 amplitudes and functional connectivity did not show any significant differences (Figures 5A,D).

In the long-time-scale comparison (Figure 6), functional connectivity in the right hemisphere in the good recall trials was significantly stronger than that in the poor recall trials: *t*(9) = 4.041, *p* < 0.01 (Figures 6E–G). The P300 amplitudes, spectral power bands and their ratios, and pupillary changes did not show any significant differences (Figures 6A–D).

The overall results of our statistical analysis of the physiological responses are shown in Table 1. Significant differences in physiological responses were extracted from the P300 amplitudes of the EEG signals in the short-time-scale comparison, from pupillary dilation in the pupil diameter signals in the middle-time-scale comparison, and from functional connectivity in the NIRS signals in the long-time-scale comparison.

**TABLE 1:** Statistical results of multiple-time-scale comparison of physiological responses extracted from EEG, NIRS, and pupil diameter signals based on task scores and serial positions in relation to attention.

**Time scale**	**Comparison items**	**Physiological responses**		
		
		**EEG**	**NIRS**	**Pupil diameter**
Short	Word recall success or fail	Significant (*p* < 0.05)	NS	NS
Middle	Serial position group	NS	NS	Significant (*p* < 0.01)
Long	Number of words recalled	NS	Significant (*p* < 0.05)	NS

*NS, not significant.*

## Discussion

### Response to Serial Position Task

#### Success Rate

The results showed that there was a significant dependence of the success rate for recalling words on the serial position (Figure 3A) as had been found in previous studies (Sederberg et al., 2006; Azizian and Polich, 2007; Serruya et al., 2014). This supports our assumption that the serial position of a word affects the success rate, which reflects attention.

In comparison with previous studies, there were fewer significant differences in success rates among serial positions. Whereas various factors such as linguistic characteristics, number of presented words, and durations of memorization and recall should affect the success rate, we speculate that the mental load for controlling blink greatly suppressed the effect of serial position. To measure the pupil diameter, the participants wore goggles fitted with two cameras and were asked not to blink except during the blink phase. Therefore, the effect of the mental load for focusing attention on not blinking during memorization could eliminate the ability to mentally rehearse the word list. This is a reasonable possibility because the success rate for the last serial position group was not particularly high, indicating that the recency effect was wiped out by the cognitive load. To better understand the effects of controlling blink, further experiments with various durations of the blink phase are needed.

#### Physiological Responses

The physiological responses to the attention task were clearly extracted, and P300 was clearly observed in the EEG signals (Figure 3B), as in free recall tasks in previous studies (Azizian and Polich, 2007; Nogueira et al., 2015). The functional connectivity in NIRS signals and the changes in pupil diameter signals during a free recall task were evaluated for the first time in this study. Analysis of the NIRS signals revealed strong functional connectivity in the prefrontal and parietal regions (Figures 3F,G). This is consistent with the finding in previous studies that the attention function is related to the prefrontal and parietal regions of the brain (Uddin et al., 2010; Nagashima et al., 2014). Moreover, the change in pupil diameter during word presentation was extracted (Figure 3C) as it was in response to memory and attention tasks in previous studies (Murphy et al., 2011; Wierda et al., 2012).

### Comparison of Physiological Responses by Multiple-Time-Scale Analysis

To the best of our knowledge, this is the first study using multiple-time-scale analysis of attention with a multimodal measurement of EEG, NIRS, and pupil diameter signals. The results revealed significant differences related to attention in different-time-scale comparisons depending on the mode. Significant differences were extracted from the P300 amplitudes in the short-time-scale comparison (1 s), pupillary dilation in the middle-time-scale comparison (10 s), and functional connectivity in the long-time-scale comparison (44.2 s). It should be noted that these results do not prove that non-significant physiological responses do not work for attention evaluation. Therefore, it cannot be directly recommended to use EEG signals for short time-scale, pupil diameter signals for middle time scale and NIRS signals for long time-scale. Nevertheless, these results suggest that the responses extracted from different physiological signals carry different types of information useful for evaluating attention. Thus, applications requiring objective attention evaluation should be developed in consideration of these different temporal characteristics of physiological responses.

In the short-time-scale comparison, a significant difference was extracted between the recall and fail words from only the P300 amplitudes (Figures 4A,B). This is consistent with the finding in previous studies that event-related potential differs between recall and fail words (Azizian and Polich, 2007). Considering that the P300 amplitudes did not show any significant differences in the middle- and long-time-scale comparisons, the P300 amplitude apparently reflects instantaneous attention rather than longer attention.

In the middle-time-scale comparison, only pupillary dilation showed significant differences among serial position groups (Figures 5B,C). Pupil diameter is regulated by the autonomic nervous system (sympathetic and parasympathetic nervous systems). The change in pupil diameter is an indicator of activity in the locus coeruleus (Sterpenich et al., 2006; Gilzenrat et al., 2010; Nagashima et al., 2014), which includes noradrenergic neurons, and the reaction time of sympathetic nervous activity of noradrenergic neurons to the organs matches the middle time scale. Therefore, significant differences in pupil diameter may reflect differences in activity in the locus coeruleus in relation to attention. It should be noted that the pupillary dilation showed significant differences between the middle position group and the last position group although the recency effect was not clearly observed in the success rates. These results indicate not only the possibility of high sensitivity of pupillary dilation for attention evaluation but also the possibility that other factors affect pupillary dilation in the later serial position groups. For example, the effect of memory retention could increase the mental load along with the effect of serial word presentation, which would increase sympathetic nervous activity. In addition, as well as the success rates, the mental load for avoiding blinking could increase sympathetic nervous activity. To better understand the effects of controlling blink and pupil diameter, further experiments with various durations of the memorization and/or blink phase are effective. When ranging the durations of the memorization and/or blink phase, deconvolution analysis (Wierda et al., 2012; Zénon, 2017) and/or components extraction (Peysakhovich et al., 2017) can be useful approaches with pupil diameter signals. Moreover, although the presented word was randomized among participants and trials and differences of screen luminance should not strongly affect the comparison based on task scores and serial position, the rapid change of screen luminance should induce the pupil constriction. Therefore, controlling the screen luminance or replacing the blank screen by a mask of an equal luminance while changing the words were desirable for future work. Thus, further experiments and analysis are needed for attention evaluation by using pupil diameter.

In the long-time-scale comparison, only functional connectivity in the right hemisphere differed significantly with the number of recall words (Figure 6G). Thus, functional connectivity enables attention to be evaluated on a long time scale. In accordance with previous studies showing that attention function is especially related to regions in the right hemisphere (Posner and Rothbart, 2007; Murphy et al., 2011), the difference in attention was extracted from the differences in functional connectivity for the right hemisphere. Whereas functional connectivity during a period of less than 1 min showed a significant difference, functional connectivity has been mainly evaluated during periods of several minutes in previous studies (Sasai et al., 2011, 2012; Tong et al., 2012). Therefore, it can be expected to be an indicator of attention on a longer time scale.

Although the different temporal characteristics of the physiological responses related to attention were suggested in this study, they were evaluated only during a specific task. Since the free recall task is an attention task in related with memory (Camos et al., 2018; Loaiza and Halse, 2019), further research is needed for evaluating various types of attention. To develop effective applications and techniques for evaluating attention, the effects of the amount and contents of the information presented, the person’s state, and the external environment should also be considered. To better understand these factors and to identify appropriate physiological responses for evaluating attention, experiments with various attention tasks should be performed. For example, the effects of the amount and contents of the information presented on the physiological responses related to attention could be evaluated by varying the number and type of words in a free recall task or by using other sustained attention tasks such as a reading span task or a trail making task (Unsworth et al., 2005; Hagen et al., 2014).

## Conclusion

Physiological responses with respect to attention during a free recall task were extracted using multimodal measurement of EEG, NIRS, and pupil diameter signals and multiple-time-scale analysis. Significant differences in physiological responses were extracted from the EEG signals in a short-time-scale comparison, from pupil diameter signals in a middle-time-scale comparison, and from NIRS signals in a long-time-scale comparison. Therefore, applications requiring objective attention evaluation should be developed in consideration of the temporal characteristics of the physiological responses.

## Data Availability Statement

The datasets generated in this study are available from the corresponding author on reasonable request.

## Ethics Statement

This study was carried out in accordance with the recommendations of the standards of the internal review board of the Research & Development Group, Hitachi, Ltd., with written informed consent from all subjects. All subjects gave written informed consent in accordance with the Declaration of Helsinki. The protocol was approved by the internal review board of the Research & Development Group, Hitachi, Ltd.

## Author Contributions

TN designed and conducted the experiments, analyzed the results, and drafted the manuscript. MK and HS designed the experiments, helped with the data analysis, and edited the manuscript.

## Conflict of Interest

TN, MK, and HS were employed by Hitachi, Ltd. The authors declare that this study received funding from Hitachi, Ltd. The funder had no role in study design, data collection and analysis, decision to publish, or preparation of the manuscript.
